# Flavin-Containing Monooxygenase 3 (FMO3) Is Critical for Dioxin-Induced Reorganization of the Gut Microbiome and Host Insulin Sensitivity

**DOI:** 10.3390/metabo12040364

**Published:** 2022-04-18

**Authors:** William Massey, Lucas J. Osborn, Rakhee Banerjee, Anthony Horak, Kevin K. Fung, Danny Orabi, E. Ricky Chan, Naseer Sangwan, Zeneng Wang, J. Mark Brown

**Affiliations:** 1 Department of Cardiovascular and Metabolic Sciences, Cleveland Clinic, Lerner Research Institute, Cleveland, OH 44195, USA; masseyw@ccf.org (W.M.); osbornl@ccf.org (L.J.O.); banerjr2@ccf.org (R.B.); horaka2@ccf.org (A.H.); fungk2@ccf.org (K.K.F.); orabid@ccf.org (D.O.); wangz2@ccf.org (Z.W.); 2 Center for Microbiome & Human Health, Cleveland Clinic, Lerner Research Institute, Cleveland, OH 44195, USA; sangwan@ccf.org; 3 Department of Molecular Medicine, Case Western Reserve University, Cleveland, OH 44106, USA; 4 Department of General Surgery, Cleveland Clinic, Cleveland, OH 44195, USA; 5 Institute for Computational Biology, Case Western Reserve University, Cleveland, OH 44106, USA; erc6@case.edu; 6 Microbial Sequencing & Analytics Core Facility, Cleveland Clinic, Lerner Research Institute, Cleveland, OH 44195, USA

**Keywords:** microbiome, diet, diabetes, pollutant, dioxin

## Abstract

Exposure to some environmental pollutants can have potent endocrine-disrupting effects, thereby promoting hormone imbalance and cardiometabolic diseases such as non-alcoholic fatty liver disease (NAFLD), diabetes, and cardiorenal diseases. Recent evidence also suggests that many environmental pollutants can reorganize the gut microbiome to potentially impact these diverse human diseases. 2,3,7,8-Tetrachlorodibenzo-p-dioxin (TCDD) is among the most potent endocrine-disrupting dioxin pollutants, yet our understanding of how TCDD impacts the gut microbiome and systemic metabolism is incompletely understood. Here, we show that TCDD exposure in mice profoundly stimulates the hepatic expression of flavin-containing monooxygenase 3 (*Fmo3*), which is a hepatic xenobiotic metabolizing enzyme that is also responsible for the production of the gut microbiome-associated metabolite trimethylamine N-oxide (TMAO). Interestingly, an enzymatic product of FMO3 (TMAO) has been associated with the same cardiometabolic diseases that these environmental pollutants promote. Therefore, here, we examined TCDD-induced alterations in the gut microbiome, host liver transcriptome, and glucose tolerance in *Fmo3*^+/+^ and *Fmo3*^−/−^ mice. Our results show that *Fmo3* is a critical component of the transcriptional response to TCDD, impacting the gut microbiome, host liver transcriptome, and systemic glucose tolerance. Collectively, this work uncovers a previously underappreciated role for *Fmo3* in integrating diet–pollutant–microbe–host interactions.

## 1. Introduction

Continuous technological advancement since the industrial revolution has brought an overall improved quality of life for modern society. However, many industrial processes often have unintended environmental consequences that negatively impact human health. Epidemiological and experimental studies of known environmental pollutants can play causal roles in various diseases including obesity and diabetes [1,2,3,4], atherosclerosis [5,6], diverse cancers [7,8,9,10], and kidney disease [11,12,13]. Many environmental pollutants can accumulate in biological systems due to inefficient turnover or breakdown of these synthetic chemicals. Upon extended exposure, environmental pollutants are sensed by diverse mammalian receptors that coordinate programs of drug detoxification or excretion. Many environmental pollutants can activate the aryl hydrocarbon receptor (AhR), which is a xenobiotic-sensing nuclear hormone receptor that initiates transcriptional programs involved in epithelial barrier function, drug detoxification, and systemic metabolic control [14,15]. The most well-known AhR response genes encode cytochrome P450 enzymes, *Cyp1a1*, *Cyp1a2*, and *Cyp1b1*. These enzymes are heme-dependent monooxygenases that participate in phase I xenobiotic metabolism, making hydrophobic compounds more soluble or better able to be conjugated by phase II metabolic processes for excretion [16]. The dioxin pollutant 2,3,7,8-Tetrachlorodibenzo-p-dioxin (TCDD) is among the most potent AhR ligand known and has been shown to bioaccumulate and exhibit endocrine-disrupting properties. In fact, TCDD-mediated activation of AhR has been linked to dioxin-driven diseases such as obesity and diabetes [1,2,3,4], atherosclerosis [5,6], diverse cancers [7,8,9,10], and kidney disease [11,12,13]. In the case of TCDD and other polycyclic aromatic hydrocarbons (PAHs), the xenobiotic metabolism process is somewhat futile in that, despite these responses, excretion is limited, and the compounds accumulate in tissue, having a half-life of up to 10 years in humans [9,17].

In addition to the well-characterized enhanced expression of cytochrome P450 enzymes in response to the TCDD–AhR signaling axis, there have been multiple reports demonstrating the transcriptional upregulation of flavin-containing monooxygenase 3 (FMO3) upon dioxin exposure [14,18]. Although FMO3 is generally upregulated upon xenobiotic exposure and plays a role in certain xenobiotic metabolic pathways and protein folding [19], it is also the enzyme responsible for the production of a gut microbe-associated metabolite called trimethylamine N-oxide (TMAO) [20,21,22,23,24]. Much like the potent AhR ligand TCDD, other PAHs such as polychlorinated biphenyl (PCB)126 and PCB77 have been shown to induce FMO3 and secondarily increase plasma TMAO [5,24]. It is interesting to note that similar to environmental pollutant exposure, the expression of FMO3 and its gut microbiome-associated enzymatic product TMAO have been associated with a wide variety of human diseases, including atherosclerosis [20,22,25], diabetes and obesity [20,21,26], kidney disease [27,28,29,30,31], and neurodegenerative disease [32,33,34,35,36]. Given the fact that FMO3 expression can be stimulated by AhR ligands and environmental pollutants [14,18,24], and the fact that the TMAO pathway has also been linked to cardiometabolic disease [20,21,22,23], we hypothesized that FMO3 may contribute to TCDD-mediated toxicity and metabolic reorganization. To investigate this hypothesis, we examined the effects of TCDD on the gut microbiome, host transcriptome, and glucose tolerance in wild-type (*Fmo3*^+/+^) or global *Fmo3* knockout (*Fmo3*^−/−^) mice. Our results demonstrate that TCDD-induced alterations in the gut microbiome, host liver transcriptome, and systemic glucose tolerance are powerfully shaped by the xenobiotic-metabolizing enzyme FMO3.

## 2. Results

### 2.1. TCDD Exposure Stimulates the Expression and TMAO-Producing Function of FMO3

**Figure 1:**
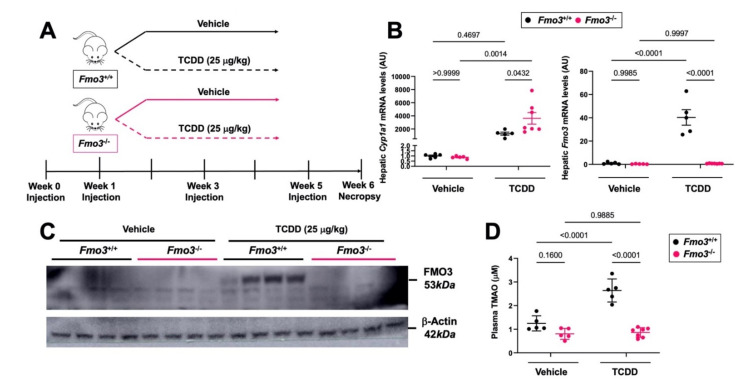
**TCDD Exposure Stimulates the Gene Expression, Protein Abundance, and TMAO-Producing Function FMO3 in Male Mice**. (**A**) Experiment schematic showing that age-matched male *Fmo3*^+/+^ or *Fmo3*^−/−^ mice were injected weekly with either corn oil vehicle or 25 µg/kg TCDD for 6 weeks. (**B**) Quantitative PCR (qPCR) analysis for the aryl hydrocarbon receptor target gene Cytochrome P450 family 1 subfamily A member 1 (*Cyp1a1*) and flavin-containing monooxygenase 3 (*Fmo3*). Data were analyzed using a 2-way ANOVA followed by a Tukey post hoc test. (**C**) Western blot of N = 4/group showing that FMO3 protein expression is elevated in *Fmo3*^+/+^ mice treated with TCDD. (**D**) Plasma trimethylamine N-oxide (TMAO) levels were quantified using stable isotope dilution liquid chromatography tandem mass spectrometry (LC-MS/MS). Data represent the mean ± S.E.M. from 5 to 7 mice per group. Data were analyzed using a 2-way ANOVA followed by a Tukey post hoc test.

It has previously been reported that exposure to dioxin-like pollutants can induce the expression of FMO3 [5,15]. However, it is not known whether FMO3 upregulation is required for the endocrine-disrupting properties of the potent dioxin TCDD. To test this, we first treated male *Fmo3* wild-type (*Fmo3*^+/+^) or knockout (*Fmo3*^−/−^) mice with weekly injections of either corn oil vehicle or TCDD (25 µg/kg) for 6 weeks (Figure 1A). After six weeks, we found that FMO3, which is not usually expressed in male mice during the light cycle [37,38], is strikingly upregulated at the mRNA (*p* < 0.0001) and protein level by TCDD treatment in wild-type mice (Figure 1B,C). Further, using plasma TMAO as a readout of FMO3 enzymatic activity, we confirmed that plasma TMAO is increased with TCDD treatment in *Fmo3*^+/+^ mice (*p* < 0.0001), and TCDD-induced increases in plasma TMAO are prevented in *Fmo3*^−/−^ mice (Figure 1D). When we examined the hepatic expression of the well-known AhR target gene Cytochrome P450 family 1 subfamily A member 1 (*Cyp1a1*), TCDD treatment caused an expected ≈2000-fold induction (*p* = 0.4697) in *Fmo3*^+/+^ mice compared to vehicle-treated mice (Figure 1B). However, TCDD treatment resulted in a ≈4000-fold induction of *Cyp1a1* in *Fmo3*^−/−^ mice (*p* = 0.0014), which was significantly increased compared to the response in *Fmo3*^+/+^ mice (*p* = 0.0432) (Figure 1B). Collectively, these results demonstrate that the hepatic expression and TMAO-producing function of FMO3 is induced by TCDD exposure, and TCDD-driven increases in hepatic AhR-target gene expression are altered in *Fmo3*^−/−^ mice (Figure 1).

### 2.2. Genetic Deficiency of Fmo3 Results in Glucose Intolerance in TCDD-Treated Mice

**Figure 2:**
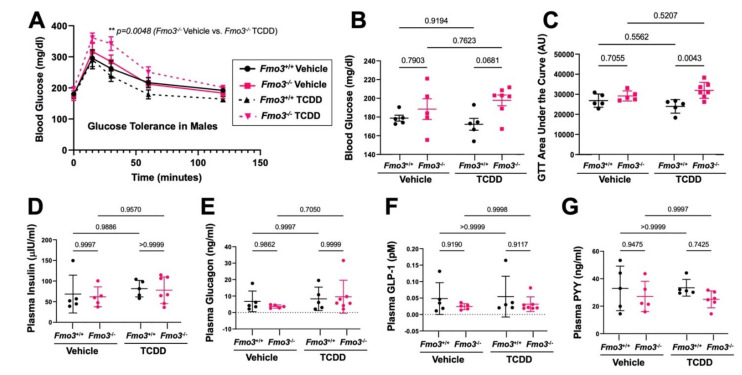
**TCDD Treatment Promotes Glucose Intolerance in *Fmo3*^−/−^ Male Mice**. Age-matched male *Fmo3*^+/+^ or *Fmo3*^−/−^ mice were injected weekly with either corn oil vehicle or 25 µg/kg TCDD for 4 weeks. (**A**) Blood glucose was measured after a 4 h fast and then 15, 30, 60, and 120 min during an intraperitoneal glucose tolerance test (GTT). Data represent the mean ± S.E.M. from 5 to 7 mice per group. Data were analyzed using a 3-way ANOVA followed by a Tukey post hoc test. (**B**) Blood glucose was measured after 4 h of fasting and revealed no significant changes due to genotype or treatment. Data represent the mean ± S.E.M. from 5 to 7 mice per group. Data were analyzed using a 2-way ANOVA followed by a Tukey post hoc test. (**C**) The area under the curve (AUC) was measured for each animal over the course of the GTT, showing that there is a significant increase in AUC in TCDD-treated KO vs. WT mice. Data represent the mean ± S.E.M. from 5 to 7 mice per group. Data were analyzed using a 2-way ANOVA followed by a Tukey post hoc test. (**D**–**G**) Levels of plasma hormones levels were quantified for (**D**) insulin, (**E**) glucagon, (**F**) active glucagon-like peptide 1 (GLP-1), and (**G**) peptide YY (PYY). Data represent the mean ± S.E.M. from 5 to 7 mice per group. Data were analyzed using a 2-way ANOVA followed by a Tukey post hoc test.

There is an abundance of evidence that exposure to dioxin-like chemicals can result in glucose intolerance and other related metabolic disturbances [1,2,3,4,5]. Given that human epidemiological and rodent studies have demonstrated that both TCDD exposure [1,2,3,4] and alterations in the FMO3–TMAO pathway [20,21,26] are associated with glucose intolerance, we subjected our mice to an intraperitoneal glucose tolerance test (GTT) after 4 weeks of TCDD exposure. Interestingly, we found that TCDD-treated *Fmo3*^−/−^ mice display clear glucose intolerance compared to TCDD-treated *Fmo3*^+/+^ mice (Figure 2A–C). Although fasting blood glucose levels were not significantly different in *Fmo3*^−/−^ mice in both treatment groups (Figure 2B), the TCDD-treated *Fmo3*^−/−^ mice have significantly higher blood glucose compared to the TCDD-treated *Fmo3*^+/+^ controls at the 30-min time point of GTT (*p* = 0.0048) (Figure 2A). Similarly, when comparing the area under the curve (AUC) during the GTT, TCDD-treated *Fmo3*^−/−^ mice had significantly elevated (*p* = 0.0043) GTT AUC compared to TCDD-treated *Fmo3*^+/+^ mice (Figure 2C). Given the well-known endocrine-disrupting properties of dioxins, we next measured plasma hormone levels and found there was no significant differences in insulin, glucagon, glucagon-like peptide 1, or peptide YY at the time of necropsy (Figure 2D–G). Together, these data suggest that the genetic deficiency of *Fmo3* does not significantly alter metabolic hormone levels, but when exposed to TCDD, male *Fmo3*^−/−^ mice exhibit marked glucose intolerance compared to TCDD-treated *Fmo3*^+/+^ controls (Figure 2).

### 2.3. TCDD-Induced Gene Expression Is Significantly Altered in the Liver of Male Fmo3^−/−^ Mice

**Figure 3:**
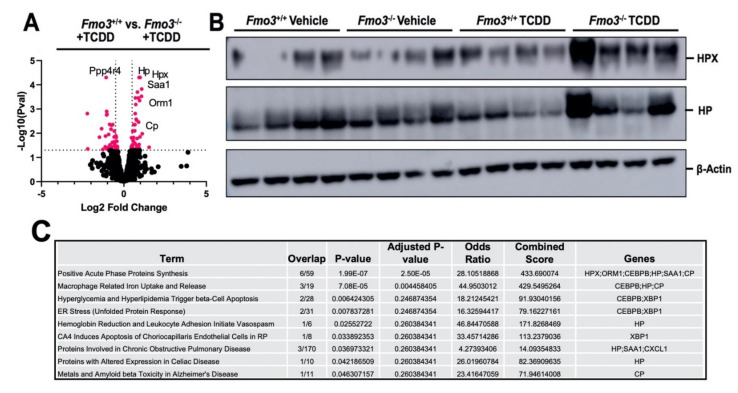
**RNA Sequencing Demonstrates that TCDD-Induced Gene Expression is Significantly Altered in Male *Fmo3*^−/−^ Mice**. (**A**) Volcano plot generated from bulk RNAseq data in *Fmo3*^+/+^ or *Fmo3*^−/−^ mice treated with TCDD. (**B**) Western blot analysis of hemopexin (HPX) and haptoglobin (HP). Data shown are from N = 4 mice per group. (**C**) Pathway analysis of 86 significantly differentially expressed genes by RNAseq using the Elsevier Pathway Collection. Sorted by *p*-value ranking.

**Figure 4:**
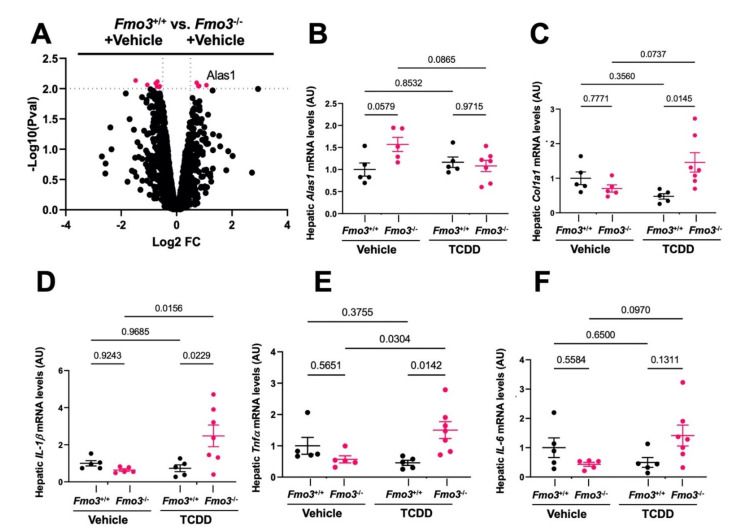
**Pro-Inflammatory and Pro-Fibrotic Gene Expression Signatures Are Altered in *Fmo3*^−/−^ mice**. (**A**) Volcano plot generated from bulk RNAseq data in *Fmo3*^+/+^ or *Fmo3*^−/−^ mice treated with corn oil vehicle; N = 4 per group. (**B**–**F**) qPCR confirmation of the most differentially expressed genes (DEGs) in mouse liver including 5′-aminolevulinate synthase 1 (*Alas1*), collagen 1a1 (*Col1a1*), interleukin 1 β (*IL-1β*), tumor necrosis factor α, (*Tnfα*), and interleukin 6 (*IL-6*). Data represent the mean ± S.E.M. from 5 to 7 mice per group. Data were analyzed using a 2-way ANOVA followed by a Tukey post hoc test.

TCDD exposure is well known to reorganize AhR target gene expression as well as other pro-inflammatory pathways in the liver to promote glucose intolerance and other related metabolic disturbances [15,39,40,41,42,43]. To better understand how *Fmo3* shapes TCDD-induced gene expression in the liver, we performed unbiased bulk RNA sequencing (RNAseq) in TCDD-treated *Fmo3*^+/+^ and *Fmo3*^−/−^ mice (Figure 3). Upon the most differentially expressed genes (DEGs) (available in Supplementary Materials), we found that *Fmo3*^−/−^ mice exhibited altered heme metabolism and acute phase gene expression versus *Fmo3*^+/+^ mice (Figure 3). The heme metabolism genes upregulated in TCDD-treated *Fmo3*^−/−^ mice were ceruloplasmin (*Cp*, *p* = 0.0044), hemopexin (*Hpx*, *p* = 5 × 10^−5^), and haptoglobin (*Hp*, *p* = 5 × 10^−5^). *Cp* is a copper containing oxidase that oxidizes iron and thus is critical for preventing oxidative damage caused by reduced iron [44]. *Hp* and *Hpx* are scavengers of hemoglobin and heme, which are also critical in the prevention of iron-related redox stress [45,46]. It is especially intriguing that these heme metabolism genes are upregulated given that the classical AhR taget gene and heme-dependent oxygenase, *Cyp1a1*, is increased in TCDD-treated *Fmo3*^−/−^ mice relative to *Fmo3*^+/+^ mice (Figure 1B). In addition to these acute phase response genes that regulate heme metabolism, other pro-inflammatory acute phase genes including serum amyloid A 1 (*Saa1*, *p* = 1.5 × 10^−4^) and orosomucoid 1 (*Orm1*, *p* = 4.5 × 10^−4^) were upregulated in TCDD-treated *Fmo3*^−/−^ mice (Figure 3). *Saa1* and *Orm1* have been implicated in altering metabolic pathways via insulin and leptin receptor signaling, respectively [47,48]. Interestingly, *Orm1* also relates to heme metabolism too in that it interacts with the hemoglobin beta chain [49]. In addition to the dysregulation of acute phase heme related pathways, the protein phosphatase 4 regulatory subunit 4, *Ppp4r4*, was downregulated in TCDD-treated *Fmo3*^−/−^ mice when compared to TCDD-treated *Fmo3*^+/+^ mice (*p* = 5 × 10^−5^). *Ppp4r4* has been identified to complex with Protein Phosphatase 4 (PP4) independently of other regulatory subunits [50]. It is interesting to note that PP4 has been implicated in immune signaling and insulin resistance [51,52,53]. Under corn oil vehicle treatment, KO mice displayed slightly increased 5′-aminolevulinate synthase 1 (*Alas1*) expression (*p* = 0.0005 (RNAseq), *p* = 0.0579 (qPCR)) (Figure 4A,B), which was recently found to be required for the AhR-mediated induction of *Cyp1a1* by PAHs and is part of the heme biosynthesis pathway [39]. Additionally, TCDD-treated *Fmo3*^−/−^ mice had elevated expression of pro-inflammatory (*TNFα* *p*= 0.0142, *IL-1* *β* (*p* = 0.0229)) and pro-fibrotic (*Col1a1* (*p* = 0.0145)) genes compared to TCDD-treated *Fmo3*^+/+^ mice (Figure 4C–F).

### 2.4. TCDD-Driven Reorganization of the Gut Microbiome Is Altered in Male Fmo3^−/−^ Mice

**Figure 5:**
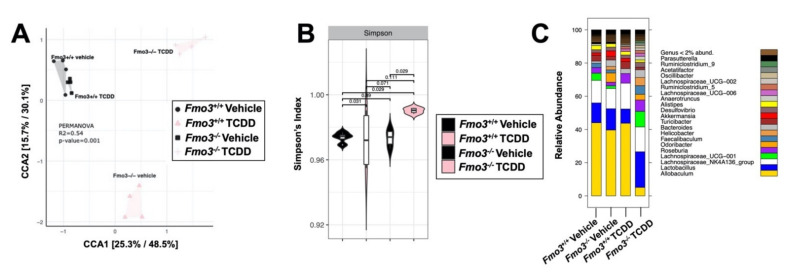
**TCDD treatment significantly alters gut microbiota in an Fmo3-dependent manner**. 16S rRNA gene sequencing was conducted in cecal samples from vehicle and TCDD-treated *Fmo3*^+/+^ and *Fmo3*^−/−^ mice. (**A**) Beta diversity was analyzed using the Bray–Curtis dissimilarity matrix and plotted using non-metric multidimensional scaling (NMDS). PERMANOVA analysis shows that there are significant differences between groups. N = 4/group (**B**) Alpha diversity was measured with the Shannon Index and shows that TCDD-treated KO mice have significantly (*p* < 0.05, Mann–Whitney *U* test) higher alpha diversity than TCDD-treated WTs and corn oil-treated KOs. N = 4/group. (**C**) The cumulative relative abundance of the most abundant microbial genera (>2%, not rarefied). Each stacked color bar represents a different genus. Total diversity patterns revealed that TCDD-treated KO mice show significant reductions in *Akkermansia* and *Allobaculum* compared to all other groups. N = 4/group.

**Figure 6:**
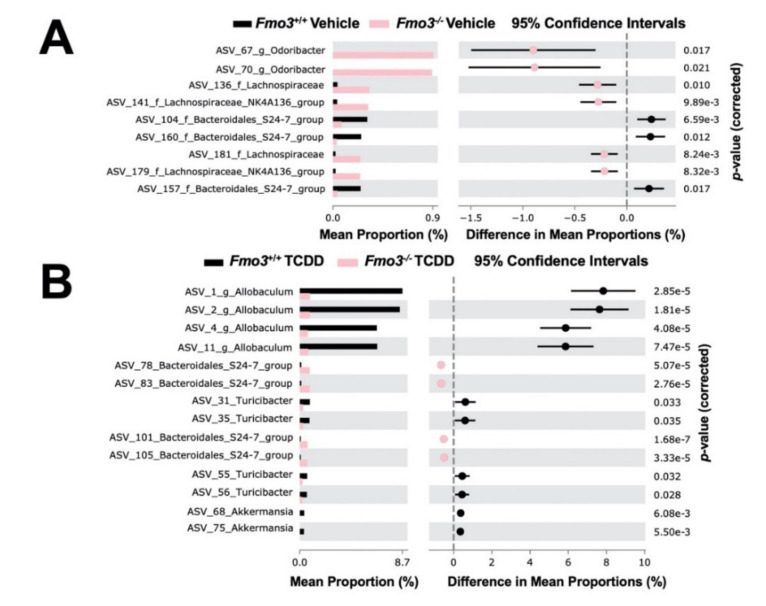
**TCDD treatment significantly alters gut microbiota in an *Fmo3*-dependent manner**. The 16S rRNA gene sequencing was conducted in cecal samples from vehicle and TCDD-treated *Fmo3*^+/+^ and *Fmo3*^−/−^ mice. ASVs significantly different in abundance (White’s non-parametric *t*-test with Benjamini–Hochberg FDR multiple test correction, adjusted *p* ≤ 0.01). (**A**) Significantly different ASVs when comparing *Fmo3*^+/+^—vehicle treated versus *Fmo3*^+/+^—TCDD-treated. (**B**) Significantly different ASVs when comparing *Fmo3*^+/+^—TCDD-treated versus *Fmo3*^−/−^—TCDD-treated.

**Figure 7:**
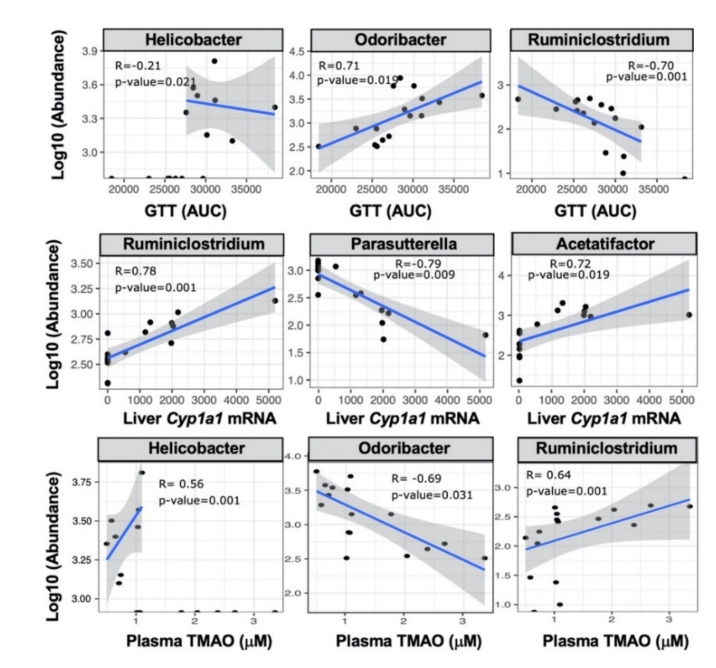
**Gut Microbiome Alterations Correlate with Host Phenotypes**. The 16S rRNA gene sequencing was conducted in cecal samples from vehicle and TCDD-treated *Fmo3^+/+^* and *Fmo3^−/−^* mice. Linear regression (parametric) and Wilcoxon (non-parametric) tests were performed on amplicon sequence variant abundances against meta-data variable levels using their base functions in R. The correlations shown are analyses comparing the abundance of ASVs and key phenotypes under investigation including glucose tolerance test area under the curve—GTT (AUC), liver cytochrome P450 family 1 subfamily A member 1 (*Cyp1a1)* messenger RNA (mRNA) expression, and plasma trimethylamine N-oxide (TMAO) levels.

Given the reported role of the microbiota in TCDD-induced toxicity and metabolic disturbance [54], we performed 16S rRNA gene sequencing in cecal samples from vehicle and TCDD-treated *Fmo3*^+/+^ and *Fmo3*^−/−^ mice (Figure 5). We analyzed beta diversity by the Bray–Curtis dissimilarity index and observed distinct clusters of both genotype-specific and treatment-specific separations, indicating that there are significant changes in the gut microbial community with changes when comparing *Fmo3* alterations driven by both FMO3 and TCDD treatment (Figure 5A). When analyzing alpha diversity by the Shannon index, it becomes apparent there is a significant increase in microbial diversity in the TCDD-treated *Fmo3*^−/−^ mice specifically (Figure 5B), which has been controversially linked to both improved health [55,56,57] and worsened conditions [58,59]. TCDD-treated *Fmo3*^−/−^ mice show near complete losses in *Akkermansia* and *Allobaculum* (Figure 5C). Of note, Akkermansia and Allobaculum have been reported as protective against glucose intolerance [38,60,61]. When comparing microbiomes in vehicle-treated *Fmo3*^+/+^ versus *Fmo3*^−/−^ mice (Figure 6A), we found that a total of 9 amplicon sequence variants (ASVs) significantly differed in abundance (White’s non-parametric *t*-test with Benjamini–Hochberg FDR multiple test correction, adjust *p* ≤ 0.01). ASVs that decreased in abundance in vehicle-treated, *Fmo3*^−/−^ mice compared to *Fmo3*^+/+^ mice were members of the *Bacteroidales_S24-7* group, whereas genera that were inceased in *Fmo3*^−/−^ mice included members of the *Odoribacter* and *Lachnospiraceae* groups. When comparing the cecal microbial community in TCDD-treated *Fmo3*^+/+^ versus *Fmo3*^−/−^ mice (Figure 6B), we found that a total of 14 ASVs significantly differed in abundance. ASVs that decreased in the TCDD-treated *Fmo3*^−/−^ mice compared to *Fmo3*^+/+^ mice were members of the genera *Allobaculum*, *Turicibacter*, and *Akkermansia*, whereas genera that were increaed in *Fmo3*^−/−^ mice included members of *Bacteroidales*. We next analyzed how the alterations in cecal microbial relative abundance correlated with key phenotypes under investigation here including GTT AUC, liver *Cyp1a1* mRNA expression, and plasma TMAO levels (Figure 7). These correlation analyses showed that the relative abundance of *Odoribacter* and *Ruminoclostridium* were most significantly correlated with both GTT AUC and plasma TMAO levels. Furthermore, the relative abundance of *Ruminoclostridium*, *Parasutterella*, and *Acetatifactor* were strongly correlated with the hepatic expression of the AhR target gene *Cyp1a1* (Figure 7). Collectively, these results demonstrate that the ability of TCDD to shape the microbiome is dramatically altered in *Fmo3*^−/−^ mice.

### 2.5. The Effects of TCDD Are Also Altered in Female Fmo3^−/−^ Mice

**Figure 8:**
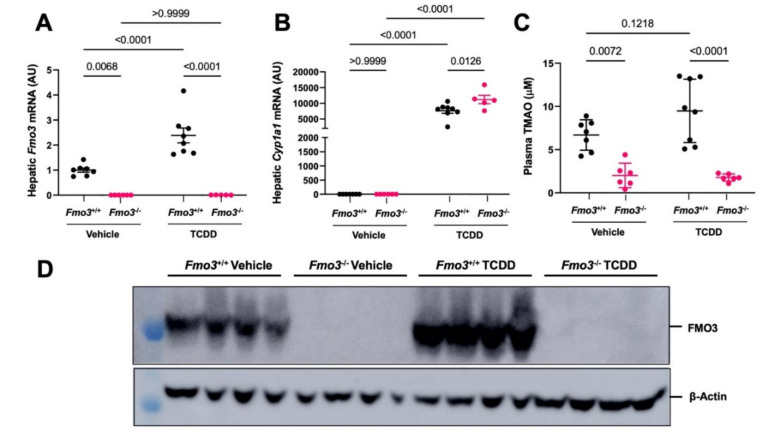
**The Effects of TCDD Are Also Altered in Female *Fmo3*^−/−^ Mice**. Age-matched female *Fmo3*^+/+^ or *Fmo3*^−/−^ mice were injected weekly with either corn oil vehicle or 25 µg/kg TCDD for 6 weeks. (**A**–**B**) Quantitative PCR (qPCR) analysis for the aryl hydrocarbon receptor target gene Cytochrome P450 family 1 subfamily A member 1 (*Cyp1a1*) and flavin-containing monooxygenase 3 (*Fmo3*). (**C**) Plasma trimethylamine N-oxide (TMAO) levels. (**D**) Western blot of liver lsyates. N = 4 per group. All other data represent the mean ± S.E.M. from 5 to 7 mice per group. Data were analyzed using a 2-way ANOVA followed by a Tukey post hoc test.

**Figure 9:**
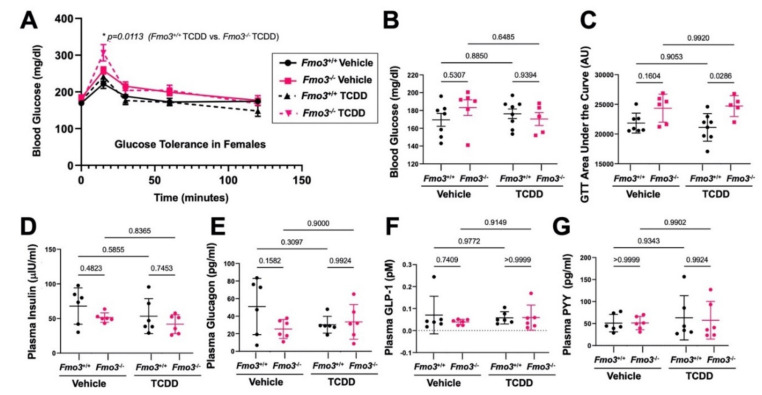
**Genetic Deficiency of *Fmo3* Impairs Glucose Tolerance in a TCDD-Dependent Manner in Female Mice**. Age-matched female *Fmo3*^+/+^ or *Fmo3*^−/−^ mice were injected weekly with either corn oil vehicle or 25 µg/kg TCDD for 4 weeks. (**A**) Blood glucose was measured after a 4 h fast and then 15, 30, 60, and 120 min during an intraperitoneal glucose tolerance test (GTT). Data represent the mean ± S.E.M. from 5 to 8 mice per group. Data were analyzed using a 3-way ANOVA followed by a Tukey post hoc test. (**B**) Blood glucose was measured after 4 h of fasting. Data represent the mean ± S.E.M. from 5 to 8 mice per group. Data were analyzed using a 2-way ANOVA followed by a Tukey post hoc test. (**C**) The area under the curve (AUC) was measured for each animal over the course of the GTT. Data represent the mean ± S.E.M. from 5 to 8 mice per group. Data were analyzed using a 2-way ANOVA followed by a Tukey post hoc test. (**D**–**G**) Levels of plasma hormones levels were quantified for (**D**) insulin, (**E**) glucagon, (**F**) active glucagon-like peptide 1 (GLP-1), and (**G**) peptide YY (PYY). Data represent the mean ± S.E.M. from 5 to 8 mice per group. Data were analyzed using a 2-way ANOVA followed by a Tukey post hoc test.

It is well appreciated that in C57BL/6 mice, there is a striking sexual dimorphism in the hepatic expression of *Fmo3*, where females have much higher basal expression compared to males [37]. Given the clear differences in basal Fmo3 expression in male versus females, we also treated female *Fmo3* wild-type (*Fmo3*^+/+^) or knockout (*Fmo3*^−/−^) mice with weekly injections of either corn oil vehicle or TCDD (25 µg/kg) for 6 weeks (Figure 8 and Figure 9). Much like we saw in male mice (Figure 1), we found that *Fmo3* is upregulated at the mRNA and protein level by TCDD treatment in wild-type female mice (Figure 8A,D). However, unlike males, TCDD treatment in females did not result in a significant elevation in plasma TMAO in *Fmo3*^+/+^ mice (Figure 8C). However, female *Fmo3*^−/−^ mice had much lower TMAO levels compared with *Fmo3*^+/+^ mice in both vehicle and TCDD treatment conditions (Figure 8C). When we examined the hepatic expression of *Cyp1a1*, we saw that when exposed to TCDD, *Fmo3*^−/−^ mice showed a significantly increased level of *Cyp1a1* expression compared to *Fmo3*^+/+^ mice, which is consistent with what we observed in male mice (Figure 1B). In female mice, we also found that glucose homeostasis was altered upon TCDD exposure in *Fmo3*^−/−^ mice (Figure 9). Upon TCDD exposure, female *Fmo3*^−/−^ mice did not have significantly elevated fasting blood glucose levels (Figure 9B). However, female *Fmo3*^−/−^ mice treated with TCDD did have significantly higher blood glucose compared to the TCDD-treated *Fmo3*^+/+^ controls at the 15 min time point of GTT (Figure 9A) and a significantly higher GTT AUC response compared to Fmo3^+/+^ TCDD-treated mice (Figure 9C). We next measured plasma hormone levels and found there was no significant differences in insulin, glucagon, glucagon-like peptide 1, or peptide YY in female mice (Figure 9D–G). Together, these data suggest that the genetic deficiency of *Fmo3* does not significantly alter metabolic hormone levels, but when exposed to TCDD, both male and female *Fmo3*^−/−^ mice exhibit dysregulated glucose homeostasis.

## 3. Discussion

**Figure 10:**
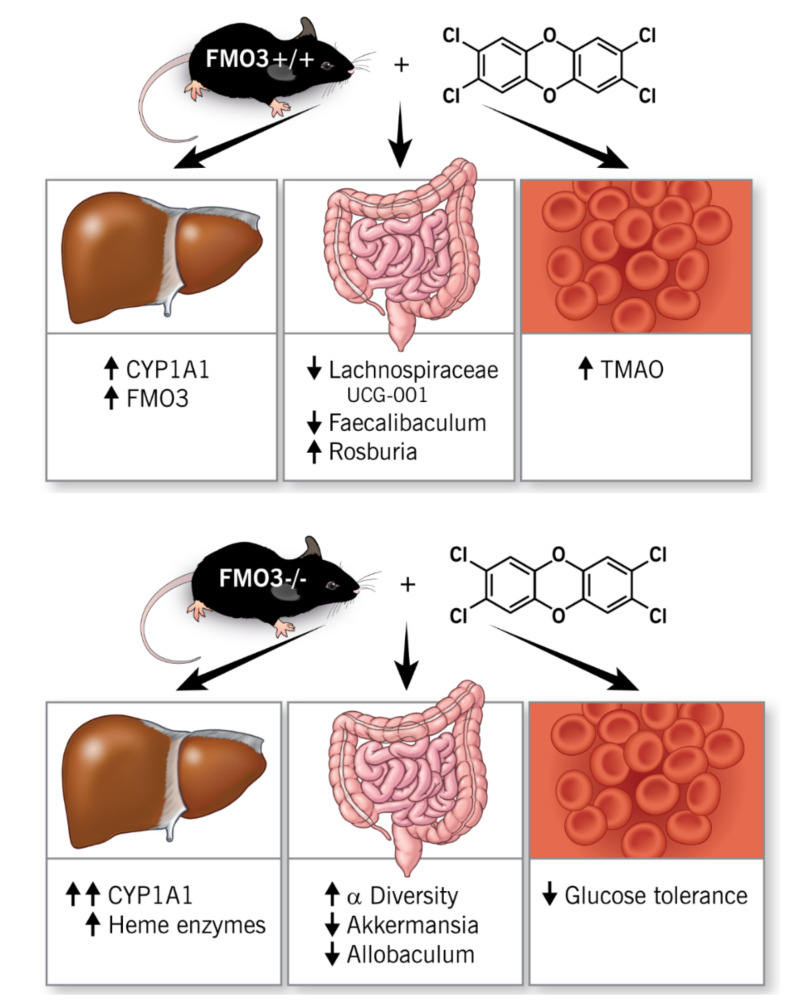
**Summary of Findings**. Collectively, the mRNA, protein, and TMAO-producing function of FMO3 are potently stimulated by TCDD exposure in mice. Both male and female *Fmo3*^−/−^ mice exhibit glucose intolerance when exposed to TCDD, despite normal fasting metabolic hormone levels. Furthermore, TCDD exposure reorganizes the gut microbiome, and TCDD-driven microbiome reorganization is dramatically altered in *Fmo3*^−/−^ mice. In addition, TCDD-driven expression of the AhR target (i.e., *Cyp1a1*), heme metabolism, pro-inflammatory, and pro-fibrotic genes is dysregulated in *Fmo3*^−/−^ mice.

Here, we have investigated the role of FMO3 in TCDD-driven reorganization of the gut microbiome and host insulin sensitivity. The key findings of this study (Figure 10) are: (1) the mRNA, protein, and TMAO-producing function of FMO3 are potently stimulated by TCDD exposure in mice; (2) both male and female *Fmo3*^−/−^ mice exhibit glucose intolerance when exposed to TCDD, despite normal fasting metabolic hormone levels; (3) TCDD exposure reorganizes the gut microbiome, and TCDD-driven microbiome reorganization is dramatically altered in *Fmo3*^−/−^ mice; and (4) TCDD-driven expression of AhR target (i.e., *Cyp1a1*), heme metabolism, pro-inflammatory, and pro-fibrotic genes is dysregulated in *Fmo3*^−/−^ mice. Interestingly, with respect to findings (3) and (4), recent studies have linked TCDD to profound effects on the microbial metabolome [62] and fibrotic responses in the liver as a result of perturbed iron homeostasis [63]. Collectively, this work demonstrates an underappreciated connection between environmental pollutants, the gut microbiome, and host glucose homeostasis. Given the fact that the gut microbial TMA–FMO3–TMAO pathway is initiated by ingestion of trimethylamine containing nutrients (i.e., choline, carnitine, γ-butyrobetaine, etc.), this work has important implications in our understanding of how dietary practices may converge with environmental exposures to impact cardiometabolic disease. Specifically, clinical prevention and treatment strategies for cardiometabolic disease may someday take into account patient history of environmental exposure, diet, microbial TMA production capacity, and FMO3 genotype. Indeed, there are ongoing studies to pharmacologically target FMO3 to reduce its ability to convert TMA to TMAO [64,65,66,67,68] as well as studies using non-lethal, microbe-targeting inhibitors that limit the production of TMA from dietary substrates [27,38,69,70,71,72].

AhR ligands such as TCDD have long been known to have endocrine-disrupting properties that can contribute to diverse human diseases including obesity and diabetes [1,2,3,4], atherosclerosis [5,6], various cancers [7,8,9,10], and kidney disease [11,12,13]. It is interesting to note that the TMA–FMO3–TMAO pathway has also been linked to these same diseases in humans [20,21,22,23,24,25]. This work bolsters the emerging concept that FMO3 may serve as a critical integrator of xenobiotic metabolism and cardiometabolic disease through the microbe-dependent production of TMAO. It is important to note that a recent cross-sectional study demonstrated a clear correlation between exposure to dioxin-like pollutant body levels and plasma TMAO levels in humans [24]. Furthermore, another recent study has shown that exposure to dioxin-like polychlorinated biphenyls (PCB 126 and PCB 77) can alter the expression and enzymatic activity of FMO3 in mice [5]. Independent work has also demonstrated that the hepatic expression of *Fmo3* can be stimulated by activation of the AhR pathway [14]. Here, we have shown for the first time that the AhR-driven upregulation of FMO3 activity is a critical determinant of TCDD-induced reorganization of the gut microbiome and host insulin sensitivity.

Although this work has focused on one of the most potent AhR ligands known (TCDD), additional work will be needed to test whether the TMA–FMO3–TMAO pathway contributes to the endocrine-disrupting properties of other dioxin-like or other structurally distinct classes of environmental pollutants. In our study, we have chosen to take a hepato-centric view given the literature precedent of TCDD pathology and FMO3 function in the liver [5,12,20,22,24,41,43,73,74]. Despite this precedent and focus herein, we cannot rule out the importance of FMO3 knockout on extra-hepatic tissues, such as adipose tissue, the gastrointestinal tract, and pancreas in TCDD toxicity. Future studies may determine whether the loss of FMO3 results in hepatic injury that influences alterations in gut microbial communities or vice versa and delineate the role of hepatic versus extrahepatic FMO3 in TCDD-mediated toxicity. Continuing studies may also investigate the extent to which FMO3 is critical in response to other PAHs given that FMO3 upregulation has been described with PCB compounds [5,24].

## 4. Materials and Methods

### 4.1. Reagents

2,3,7,8-Tetrachlorodibenzo-p-dioxin (TCDD) was supplied by Agilent (RPE-029) and prepared in corn oil (Sigma, St. Louis, MO, USA, C8267) at 5 µg/mL. Primers for qRT-PCR were ordered from Sigma (see Table 1 for sequences). Antibodies were supplied by various vendors (see Table 2 for details).

### 4.2. Animals

To study the effects of TCDD under conditions of whole-body genetic deficiency, global *Fmo3* knockout (*Fmo3*^−/−^) mice were generated using homologous recombination approaches in C57BL/6 C2 embryonic stem cells (ESCs) at the Case Western Reserve University mouse transgenic core. Initially, we purchased a knockout-first, reporter-tagged insertion with conditional potential (promoter-driven cassette) plasmid from the International Knockout Mouse Consortium (IKMC project 80017—targeting vector HTGR06012_12_A_7G09) and confirmed sequence fidelity. Then, this targeting vector was linearized and electroporated into C57BL/6 C2 ESCs. After positive and negative selection, clones were isolated and confirmed to have homologous recombination by complementary Southern blotting and quantitative polymerase chain reaction (qPCR) approaches. Then, several targeted clones were combined into albino blastocysts, and multiple blastocysts were injected into pseudopregnant recipient mice, resulting in several highly chimeric founder mice. These chimeras were backcrossed to C57BL/6J mice to confirm germ-line transmission of the targeted allele by PCR. Once the line was established, we further backcrossed founder mice > 10 generations into the C57BL/6J substrain before studies. FMO3 knockout and WT littermate mice were randomly assigned to either receive 25 µg/kg TCDD or volume-matched corn oil vehicle injections intraperitoneally weekly for 6 weeks.

After 4 weeks of injection with corn oil vehicle or TCDD, all mice were subject to glucose tolerance testing. For glucose tolerance testing, mice were fasted from 0900 to 1300 then injected intraperitoneally with 1 g/kg glucose. Blood glucose was measured immediately prior to injection as well as 15, 30, 60, and 120 min after injection using Accucheck glucometers.

At sacrifice, mice were fasted from 0900 to 1300 and injected with a lethal dose of Ketamine/Xylazine (150 mg/kg and 15 mg/kg, respectively). Tissues were excised and snap frozen in liquid nitrogen. Blood was collected via cardiac puncture into EDTA tubes (BD, #365974), and plasma was collected using the manufacturer’s protocol. All mice were maintained in an Association for the Assessment and Accreditation of Laboratory Animal Care, International-approved animal facility, and all experimental protocols were approved by the Institutional Animal Care and use Committee of the Cleveland Clinic.

### 4.3. RNA Isolation, RNA Sequencing, and Quantitative PCR (qPCR) Analyses

Liver tissue was homogenized in Trizol (Life Technologies, Waltham, MA, USA, 15596018), and RNA was extracted using chloroform extraction and an RNeasy Kit (Qiagen, Beverly, MA, USA). In brief, the aqueous layer from the Trizol/chloroform extraction was passed through a gDNA eliminator column and then combined with 1 volume of 70% ethanol before loading onto the RNeasy spin column (Qiagen), at which point the manufacturer’s protocol was followed. Following isolation, RNA samples were checked for quality and quantity using Nanodrop Spectrophotometry for qPCR and the Bio-analyzer for RNAseq (Agilent, Santa Clara, CA, USA). RNA-SEQ libraries were generated using the Illumina mRNA TruSEQ Directional library kit and sequenced using an Illumina HiSEQ4000 (both according to the Manufacturer’s instructions). RNA sequencing was performed by the University of Chicago Genomics Facility.

Raw sequencing data in the form of fastq files were transferred to and analyzed by the Bioinformatics Core at Case Western Reserve University. Fastq files were trimmed for quality and adapter sequences using TrimGalore! (Version 0.6.5 Babraham Institute, https://github.com/FelixKrueger/TrimGalore, accessed on 28 February 2022), which is a wrapper script for CutAdapt and FastQC. Reads passing quality control were aligned to the mm10 mouse reference genome using STARAligner [75] (version 2.5.3a) guided using the GENCODE gene annotation. Aligned reads were analyzed for differential gene expression using Cufflinks [76] (version 2.2.1), which reports the fragments per kilobase of exon per million fragments mapped (FPKM) for each gene. Significant genes were identified using a significance cutoff of *p*-value < 0.05 and used as input for pathway analysis in enrichR.

Qualitative Reverse Transcriptase Polymerase Chain Reaction (qPCR) cDNA was prepared using a qScript cDNA supermix (QuantaBio, Beverly MA, USA, 95048). qPCR was carried using SYBR Fast reagents (Applied Biosystems, Waltham, MA, USA, 4385618) out on an Applied Biosystems StepOne Plus machine. Data were analyzed using the ΔΔC_T_ method, and expression was normalized such that the WT corn oil group’s expression equaled 1. Results were analyzed in Graphpad Prism 9, using two-way ANOVA and Tukey’s post hoc testing. *p* < 0.05 was considered significant.

### 4.4. Quantification of Plasma Hormone Levels

Plasma insulin, glucagon, peptide YY (PYY), and active glucagon-like peptide 1 (GLP-1) were measured using a custom MesoScale U-Plex assay using the manufacturer’s instructions (Meso Scale Diagnostics, Rockville, MD, USA). Results were analyzed in Graphpad Prism 9, using two-way ANOVA and Tukey’s post hoc testing. *p* < 0.05 was considered significant.

### 4.5. Western Blotting

Protein lysates were prepared by homogenizing liver tissue in a modified RIPA buffer as previously described [77]. First, 40 ug of protein were loaded onto 12% SDS-Polyacrylimide Gels (Invitrogen, Waltham, MA, USA). Then, the protein was transferred to 0.45 µm PVDF membranes (Thermo). Primary antibodies for Western blot were diluted 1:1000 in 5% (*w*/*v*) bovine serum albumin in TBST buffer, and secondary antibodies were diluted 1:5000 in 5% (*w*/*v*) milk/TBST. Antibodies were supplied by various vendors (see Table 2 for details). Blots were developed using ECL reagent (Amhersham), and images were captured with a GE Amhersham Imager 6000 (Software Version 1.1.1).

### 4.6. Quantification of Plasma TMAO Levels

Stable isotope dilution high-performance liquid chromatography tandem mass spectrometry (LC-MS/MS) was used for the quantification of TMAO levels as previously described [78,79]. The d9(methyl)-isotopologues, d9-TMAO, were spiked into plasma as internal standards. LC-MS/MS analyses were performed on a Shimadzu 8050 triple quadrupole mass spectrometer. TMAO and d9-TMAO were monitored using multiple reaction monitoring of precursor and characteristic product ions in positive mode as follows: m/z 76.0 → 58.1 for TMAO; and m/z 85.0 → 66.2 for d9-TMAO. Results were analyzed in Graphpad Prism 9, using two-way ANOVA and Tukey’s post hoc testing. *p* < 0.05 was considered significant.

### 4.7. Gut Microbiome Analyses

DNA was extracted from mouse cecal contents using the QIAGEN PowerSoil Pro kit using the manufacturer’s protocol. The 16S rRNA amplicon sequencing was completed for the V3–V4 region using an Illumina iSeq 100 system from mouse cecal contents. Raw 16S amplicon sequences and metadata were demultiplexed using the split_libraries_fastq.py script implemented in QIIME1.9.141 [80]. The demultiplexed fastq file was split into sample specific fastq files using the split_sequence_file_on_sample_ids.py script from QIIME1.9.141 [80]. Individual fastq files without non-biological nucleotides were processed using the Divisive Amplicon Denoising Algorithm (DADA) pipeline [81]. The output of the DADA2 pipeline (feature table of amplicon sequence variants) was processed for alpha and beta diversity analysis using the phyloseq [82] and microbiomeSeq (http://www.github.com/umerijaz/microbiomeSeq, accessed on 28 February 2022) packages in R. Alpha diversity estimates were measured within group categories using the estimate_richness function of the phyloseq package [82]. Non-metric multidimensional scaling (NMDS) was performed using the Bray–Curtis dissimilarity matrix [83] between groups and visualized by using the ggplot2 package [84]. We assessed the statistical significance (*p* < 0.05) throughout, and whenever necessary, we adjusted *p*-values for multiple comparisons according to the Benjamini–Hochberg method to control the false discovery rate (FDR) [85] while performing multiple testing on taxa abundance according to sample categories. We performed an analysis of variance among some categories while measuring the alpha diversity measures using the plot_anova_diversity function in the microbiomeSeq package (http://www.github.com/umerijaz/microbiomeSeq, accessed on 28 February 2022). Permutational multivariate analysis of variance (PERMANOVA) with 999 permutations was performed on all principal coordinates obtained during principal coordinates analysis with the ordination function of the microbiomeSeq package. Linear regression (parametric) and Wilcoxon (non-parametric) tests were performed on amplicon sequence variant abundances against meta-data variable levels using their base functions in R [86].

## 5. Conclusions

The results described herein have implications in our understanding of the complex interactions between environmental pollutant exposure, dietary inputs, host genetics, gut microbial metabolism and community structure, and the development of cardiometabolic disease. It is important to note that the TMAO pathway has become an attractive drug target for a number of human disease, and drugs that lower circulating TMAO levels are showing promise in preclinical animal models of cardiometabolic disease [27,31,38,69,70,71,72,87]. However, as this drug discovery process advances, it will be important to consider how TMAO-perturbing therapeutic strategies may impact microbe–host crosstalk in xenobiotic metabolism.

## Figures and Tables

**Table 1:** Primer Sequences Used for Quantitative Real-Time PCR.

Primer	Sequence (5′–3′)
Fmo3 F	CCCACATGCTTTGAGAGGAG
Fmo3 R	GGAAGAGTTGGTGAAGACCG
Cyp1a1 F	CTGAAGGTGGTAGTTCTTGGAG
Cyp1a1 R	CCATACATGGAAGGCATGATCTA
CycloA F	GCGGCAGGTCCATCTACG
CycloA R	GCCATCCAGCCATTCAGTC
Alas1 F	CACTGTCCGAGTCACATCATC
Alas1 R	TGATGGCCTGGACGTAGATA
Col1a1 F	ATGTTCAGCTTTGTGGACCTC
Col1a1 R	CAGAAAGCACAGCACTCGC
IL-1b F	AGTTGACGGACCCCAAAAG
IL-1b R	AGCTGGATGCTCTCATCAGG
TNFa F	CCACCACGCTCTTCTGTCTAC
TNFa R	AGGGTCTGGGCCATAGAACT
IL-6 F	GCTACCAAACTGGATATAATCAGGA
IL-6 R	CCAGGTAGCTATGGTACTCCAGAA

**Table 2:** Antibody Used for Immunoblotting.

Antibody	Vendor	Product #
FMO3	Abcam	ab126790
HPX	R&D Systems	AF7007
HP	Abcam	ab256454
β-Actin HRP	Cell Signaling	4970S
Rabbit HRP	Cell Signaling	7074S

## Data Availability

RNA sequencing data have been submitted to the NCBI GEO portal (accession GSE191138).

## Supplementary Materials

A supplementary table of differentially expressed genes is available at https://www.mdpi.com/article/10.3390/metabo12040364/s1.

## Abbreviations

AhR: aryl hydrocarbon receptor; CVD, cardiovascular disease; FMO, flavin-containing monooxygenase; Fmo3, flavin-containing monooxygenase 3; PAHs, polycyclic aromatic hydrocarbons; PCB, polychlorinated biphenyl; TCDD, 2,3,7,8-Tetrachlorodibenzo-p-dioxin; TMA, trimethylamine; TMAO, trimethylamine-N-oxide.
